# Physical job demands and related health complaints among surgeons

**DOI:** 10.1007/s00420-012-0763-7

**Published:** 2012-03-29

**Authors:** M. M. Ruitenburg, M. H. W. Frings-Dresen, J. K. Sluiter

**Affiliations:** Academic Medical Centre, Coronel Institute of Occupational Health, University of Amsterdam, PO Box 22700, 1100 DE Amsterdam, The Netherlands

**Keywords:** Musculoskeletal diseases, Occupational health, Physicians, Workload, Occupational exposure, General surgery

## Abstract

**Purpose:**

Surgeons’ poor physical health and high physical job demands might threaten good quality of care. We aimed to compare the prevalence of physical complaints of surgeons, their physical work ability and the physical job demands of surgeons with that of other hospital physicians.

**Methods:**

All medical doctors (*n* = 958) of one academic medical center were invited to complete the online questionnaire to assess the physical work ability and the prevalence of regional musculoskeletal complaints. A purposive sample of 44 surgeons and 82 other hospital physicians were systematically observed during work to quantify the physical job demands for an average working day.

**Results:**

More surgeons found their work to be physically strenuous (41 vs. 13 %, *p* < .000) and more were bothered by working in uncomfortable or exhausting postures (73 vs. 27 %, *p* < .001). Both groups reported that most of their physical complaints were in the neck (39 and 32 %) and arm regions (36 and 27 %). The majority of surgeons (86 %) and other hospital physicians (79 %) experienced difficulties coping with their job demands because of their physical state once a month or less. Compared with other hospital physicians, surgeons stand longer (4 vs. 3 h, *p* = .004) and perform fine repetitive movements longer (80 vs. 3 min, *p* < .001) during an average working day.

**Conclusions:**

Exposure to several physical job demands that are perceived as uncomfortable and exhausting and the presence of physical health complaints reduce surgeons’ work functioning.

## Introduction

Due to an aging society and a declining younger workforce, surgeons will have to work until old age. For surgeons to remain healthy on the job, it is important to provide an optimal work environment that minimizes the risk of developing physical health complaints. A relevant first step would be to gain insight into the effects of the physical demands of work on surgeons, because high physical work demands increase the risk of ill health (Lund et al. 2006). To our knowledge, no attempts have been made to quantify the physical work demands that surgeons experience during an average workday, although several studies have explored the physical demands of specific general and laparoscopic procedures (Kant et al. 1992; Berguer et al. 1997; Van Veelen et al. 2004). These studies have indicated that performing specific types of surgery can put intense physical strain on surgeons. Surgeons have fixed work postures, tend to work with the arms abducted from the trunk and unsupported, with the cervical spine flexed forward and rotated (Kant et al. 1992). A high static load is imposed on the both shoulder–neck region and the shoulder joint by this posture (Chaffin and Andersson 1984). Furthermore, surgery can require long-term, fixed low-back postures while performing very precise movements, resulting in awkward positioning of the arms, hands and fingers, which can be categorized as mild-to-moderate physical demands (Berguer et al. 1997). Although performing surgery obviously constitutes a significant part of the surgeon’s job, a surgeon’s average workday consists of performing other tasks as well, including ward rounds, surgical meetings, patient consultations and report-writing (Szeto et al. 2009). To be able to take preventive measures that keep surgeons healthy on the job, knowledge of the physical job demands that surgeons experience during an average working day is relevant.

The presence of high physical job demands is a potential threat to surgeons’ health because it may put them at risk of developing work-related musculoskeletal complaints (Stomberg et al. 2010). In general, risk factors for musculoskeletal complaints include awkward body postures, frequent repetitive movements and prolonged static head and back postures (Johnston et al. 2005). Surgeons have frequently reported complaints in the upper extremities, such as pain and stiffness in the neck, shoulders, back and lower back and thumbs (Johnston et al. 2005; Mirbod et al. 1995; Szeto et al. 2009; Sari et al. 2010). A broad range of prevalence rates of physical complaints among different body regions has been found, which may be explained by the different case definitions and time frames used to define the presence of a physical complaint. For example, some studies have asked respondents to report symptoms of any pain, while others have asked them to report feelings of numbness or stiffness. In addition, studies have differed in reporting point-of-time, annual or life-time prevalence of physical complaints. Aside from the short-term negative effects on well-being at work, the presence of musculoskeletal complaints is a known risk factor for long-term sickness absence (Oude Hengel et al. 2011; Roelen et al. 2007). Furthermore, physical complaints may affect surgeons in functioning at work (Hansson and Jensen 2004).

To be able to prevent the health and work function-related problems experienced by surgeons, more knowledge of these conditions is needed. Therefore, the first aim of this study was to quantify the physical job demands of surgeons and to compare them with the other hospital physicians who served as a reference group. The second aim of this study was to compare the prevalence of physical complaints and physical work ability of surgeons with that of other hospital physicians.

## Methods

Two methods, systematic observations and questionnaires, were used and reported separately. Data were gathered among surgeons and hospital physicians working in one academic medical center in The Netherlands. Ethical clearance was provided by the Medical Ethics Board of the Academic Medical Center for this study.

### Systematic observations at the workplace

To quantify the physical job demands of surgeons and other hospital physicians during an average workday in terms of duration, frequency and intensity, systematic observations using a hierarchical task analysis were conducted at the workplace.

#### Population

A purposive sample of medical doctors who specialized in one of three general medical specialties after university graduation, including observational (e.g., Internal Medicine), supportive (e.g., Clinical Genetics) and surgical (e.g., General Surgery) were eligible for this part of the study. The number of participating medical doctors depended on the number of observations following from the measurement strategy (see below).

#### Measurement strategy

The measurement strategy of the hierarchical task analysis was based on explorative interviews with one medical doctor of each of the 23 specialties, resulting in general information about the activities and body postures that could occur during a workday. The Task Recording and Analysis on Computer (TRAC) observation system (Frings-Dresen and Kuijer 1995) was used, which provides real-time data on the duration and frequency of activities and body postures of interest during work (“Appendix 1”).

A measurement strategy was developed to capture all apparent facets of the job for each day of a week, taking into account the variation in duration and frequency of tasks, activities and body postures. The main causes of variation were the type of patients and the medical doctors’ internship types, which are reflected, for example, in the activities engaged in during a workday. The measurement strategy resulted in 2-h observation periods that were divided over four predefined periods during a day shift: two periods in the morning and two in the afternoon. Based on the available working schedule, the observer asked medical doctors whether he was allowed to observe them for 2 h during work at a specific time period of the working day. Due to practical considerations, observation periods in the operating room lasted 4 h and were taken as two separate measurements. Observations were planned of a total of 44 General Surgery doctors, who represented the surgical specialties; 42 Internal Medicine doctors, who represented the observational specialties; and 40 medical doctors in several support specialties.

#### Observational procedure

Preceding the observations, the observers practiced the observation system until a high intra- and inter-observer intraclass correlation coefficient was obtained (ICC > .80).

To prevent inter-individual variation from being a potential confounder, medical doctors were randomly chosen based on their work schedule. After informing the staff and medical doctors that observations at the workplace would take place and permission was granted, the researcher contacted the medical doctors individually, after checking the work schedule, by sending them an e-mail request to participate in the study. Before the observation started, the researcher explained in detail how the observation would take place and explained that he would step back whenever the medical doctor or patient requested that he do so.

### Questionnaire study

An online questionnaire was used to assess the prevalence of musculoskeletal disorders among surgeons and hospital physicians to identify their self-rated work-relatedness of complaints and to identify whether their musculoskeletal disorders limited their work functioning. The frequency of discomfort that was experienced during work because of specific physical activities was also assessed.

#### Population and procedure

A total of 958 medical residents and doctors received the online questionnaire. This population included all specialists and all medical doctors in any of the 23 subspecialties. In autumn of 2009, the participants received an e-mail with information about the study followed by an e-mail with a link and a personal password that allowed them to access the online questionnaire. the response rate was 52 %.

#### Study measures

**Fig. 1:**
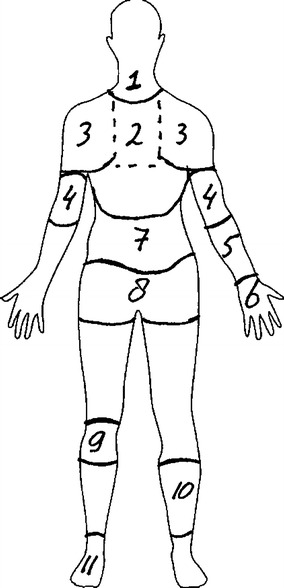
Defined body regions for reporting physical complaints (*1* = neck, *2* = upper back, *3* = shoulder, *4* = elbow, *5* = forearm, *6* = wrist, *7* = lower back, *8* = hip, *9* = knee, *10* = leg, *11* = ankle)

The questionnaire contained items designed to gather general sample information about age, gender and seniority (physician or resident). Data were also gathered concerning the prevalence of musculoskeletal disorders. The definition used for musculoskeletal disorders was regularly experienced recurrent and/or prolonged complaints in a certain body region during the past 6 months. Body regions were defined by a chart (Fig. 1). Respondents were asked to report physical complaints on both sides of their bodies. In case of a physical complaint, they were asked whether they believed that their work was (partially) responsible for developing these complaints and whether they felt impaired in executing their work because of these complaints. All questions were answered on a dichotomous scale (yes/no). The body regions of interest were neck, shoulder, upper back, elbow, forearm, wrist, lower back, hip, knee, leg and ankle.

Furthermore, a modified version of the physical demands scale of the Dutch VBBA (Van Veldhoven and Meijman 1994) was used to identify whether respondents had been seriously bothered in the past few weeks by any of several physical job demands. Responses were given on a dichotomous scale (yes/no). Concerning their physical work ability, respondents were asked to report how often during the past 3 months they had experienced difficulties in coping with their job demands because of their physical state by using a five category scale (never, once a month, several times a month, once a week, several times a week).

#### Analyses

For our first aim, the real-time data of the observations of Internal Medicine doctors and the support specialties were taken together and were considered as data representing ‘other hospital physicians’. The duration and frequency of activities and body postures from each measurement were extrapolated to an average workday of 10 h. Mean (and SD) durations and frequencies were calculated at the group level for surgeons and other hospital physicians. When primary exploration of the data revealed an average absolute duration of more than 5 min for activities and an average frequency of body postures of more than five for an average workday, they were included in the analyses. After the data were checked for normality, an appropriate analysis, depending on the type of measurement parameter, was performed to test for significant differences in means and frequencies of activities and body postures between both groups. A frequency count and a Chi-square test were performed on data regarding the subjective experience of some of the physical demands. When there were too few observations to perform a Chi-square test, the Fisher’s exact test was performed instead.

With respect to the second aim of this study, we first calculated the demographics of each group. To assess the prevalence of a musculoskeletal problem, the percentage of subjects who reported a regional complaint was calculated for each region. Within the group of respondents who reported physical complaints, the proportion who thought that work was partly responsible for developing these complaints and/or that the complaints impaired their work functioning was calculated. To test differences in the prevalence of complaints between surgeons and other hospital physicians, four body regions were formed: the neck region (neck and upper back), the lower back region, the arm region (shoulder, elbow, forearm and wrist) and the leg region (hip, knee, leg and ankle). The original response categories for physical work ability were recoded into two categories (once a month or less and several times a month or more). A frequency count and a Chi-square test were performed to test for differences. All analyses were performed using SPSS 17.0 for Windows.

## Results

All 126 of the planned observations were executed. Based on the conclusion from the explorative interviews that the tasks and activities of medical residents during a working day were the most representative of tasks and activities for a general working day, observations were performed with medical residents. From the 458 questionnaires (response rate 51 %) that were returned, a total of 395 questionnaires could be used for analysis. Some questionnaires were filled out incompletely, while a few others were filled out by medical doctors that performed non-clinical functions and were therefore considered not to be representative. Most surgeons (55 %) were males, while most of the other hospital physicians (55 %) were females (Table 1).

**Table 1:** Overview of the demographic characteristics of the questionnaire study population

	Surgeons (*n* = 100)		Hospital physicians (*n* = 295)		Total (*n* = 395)	
	%	(*n*)	%	(*n*)	%	(*n*)
Male	55	(55)	45	(131)	47	(186)
Female	45	(45)	55	(163)	53	(208)
Medical doctor	59	(59)	51	(151)	53	(210)
Medical resident	41	(41)	49	(144)	47	(185)

	Mean	(SD)	Mean	(SD)	Mean	(SD)
Age (years)	41	(10.8)	40	(9.8)	41	(10.0)

### Physical exposure

Table 2 gives an overview of the mean duration and frequency of activities and body postures. During an average working day, surgeons spent an equal amount of time sitting and standing (approximately 4 h each), whereas other hospital physicians spent more time sitting than standing (6 vs. 3 h, respectively). Surgeons make fine repetitive movements for a significantly longer time (80 min) compared with other hospital physicians (3 min), while the latter group works significantly longer on a computer (104 min) compared with surgeons (73 min). Both groups of physicians frequently perform cervical flexions or rotations, while the mean frequency of the other body postures is relatively low.

**Table 2:** Duration and frequency of activities and body postures, and a comparison between surgeons and other hospital physicians

	Surgeons (*n* = 44)		Hospital physicians (*n* = 82)		*U* ^a^	*p*
	Mean	95 % CI	Mean	95 % CI		
*Duration activities* (*min*)						
Sitting*	279	230–328	351	315–386	1,342	.018
Standing*	267	217–318	187	154–219	1,248	.004
Fine repetitive movements*	80	38–123	3	0–7	1,209	<.001
Working on a computer*	73	48–98	104	85–123	1,349	.019
Walking	45	36–54	46	41–51	1,669	.488
*Duration body postures* (*min*)						
Cervical flexion (>25°)	119	82–157	71	61–82	1,505	.125
Cervical rotation (>25°)*	27	23–32	49	39–59	1,396	.036
Lumbar flexion (>60°)	10	7–13	12	9–14	1,741	.740
Asymmetric posture	4	1–7	1	0–2	1,625	.131
Lumbar rotation (>20°)*	2	1–3	1	0–1	1,447	.003
One arm above shoulder	1	0–2	1	1–2	1,789	.902
Reaching*	1	1–2	5	3–7	1,284	.002

	Mean	Min–max	Mean	Min–max	*U*	*p*
*Frequency body postures*						
Cervical flexion (>25°)	334	85–705	315	10–965	1,616	.336
Cervical rotation (>25°)	289	70–610	410	5–1405	1,518	.143
Lumbar flexion (>60°)	36	0–105	52	0–255	1,551	.194
Reaching*	25	19–31	67	47–88	1,127	.001
Lumbar rotation (>20°)*	14	0–55	9	5–13	1,189	.001
Asymmetric posture*	13	0–135	5	0–50	1,444	.034
One arm above shoulder	9	0–60	13	0–110	1,710	.605

^a^The non-parametric Mann–Whitney *U*-test was performed on the data to investigate differences between both groups

* Difference is significant (*p* < .05)

In addition to the quantified job demands, Table 3 shows the percentage of respondents that felt seriously bothered by specific physical activities. A larger proportion of surgeons than hospital physicians found their work physically strenuous (41 vs. 14 %, respectively). In addition, a larger proportion of surgeons felt seriously bothered by making prolonged repetitive movements (35 vs. 18 %, respectively), working in uncomfortable or exhausting postures (73 vs. 27 %, respectively) and using hand tools (8 vs. 3 %, respectively).

**Table 3:** Proportion (%) of respondents who were seriously bothered by certain physical job demands, and a comparison between both groups

Physical demands	Surgeons (*n* = 90–91)		Hospital physicians (*n* = 279–280)		χ^2^	*p*
	%	(*n*)	%	(*n*)		
In your work, are you seriously bothered by….?						
…having to lift or move loads	10	(9)	9	(25)	.076	.782
…frequently have to bend down	9	(8)	9	(25)	.002	.968
…regularly having to reach up too high for objects	0	(0)	3	(9)	3.009	.120
…having to do the same movements continuously for a long period of time*	35	(32)	18	(51)	11.362	.001
…using hand tools*	8	(7)	3	(7)	5.175	.049
Do you have to work in uncomfortable or tiring positions?*	73	(66)	27	(75)	60.989	<.001
Do you find your work physically strenuous?*	41	(37)	13	(35)	34.819	<.000

* Difference is significant (*p* < .05)

### Musculoskeletal complaints

Few surgeons and few hospital physicians reported complaints in the hip, knee, leg and ankle/foot region (see “Appendix 2”). The most often reported physical complaints were located in the neck, upper and lower back and shoulder region. Except for reported physical complaints in the hip region, at least half of the surgeons who reported physical complaints framed these complaints as work-related. Furthermore, at least one of every three surgeons who reported physical complaints in the shoulder, forearm, wrist/hand and knee region indicated that these complaints impaired their work functioning. Most hospital physicians feel impaired in their work functioning by physical complaints in the forearm (43 %), leg (43 %) and elbow (42 %) regions.

Although one out of every three hospital physicians (37 %) reported having experienced physical complaints in the neck region, significantly more surgeons (50 %) reported complaints in this region (Table 4). Compared with hospital physicians, significantly more surgeons (56 vs. 14 %, respectively) indicated that their work contributed to physical complaints in the leg region. Although not statistically significant, it appears to be a trend that more surgeons compared with other hospital physicians reported their work as being a contributing factor in the development of physical complaints in the neck and lower back region. The number of surgeons and other hospital physicians who felt impaired in their work functioning due to physical complaints in the different body regions ranges from 12 to 42 %, but no significant differences were found between the two groups.

**Table 4:** Overview of the percentage (%) of respondents with physical complaints in each summed body region

Physical complaints	Surgeons (*n* = 91)		Hospital physicians (*n* = 281)		*χ* ^*2*^	*p*
	%	(*n*)	%	(*n*)		
Neck	39	(35)	32	(89)	1.426	.232
Work-related	80		69		1.629	.202
Work-impairing	17		15		.125	.724
Lower back	24	(22)	25	(69)	.005	.942
Work-related	59		38		3.122	.077
Work-impairing	18		16		.061	.805
Arm	36	(33)	27	(76)	2.819	.093
Work-related	61		63		.064	.801
Work-impairing	42		26		2.782	.095
Leg	10	(9)	18	(51)	3.466	.063
Work-related*	56		14		8.366	.004
Work-impairing	22		12		.724	.395

* Difference is significant (*p* < .05)

Table 5 shows that the majority of surgeons (86 %) and other hospital physicians (79 %) rarely experienced difficulties coping with the physical demands of their jobs because of their physical state. However, one out of every seven surgeons (14 %) and one out of every five other hospital physicians (21 %) experienced difficulties at work because of impairments in their physical well-being.

**Table 5:** How often in the past 3 months did you experience difficulties coping with the job demands because of your physical state?

	Surgeons (*n* = 93)		Hospital physicians (*n* = 284)	
	%	(*n*)	%	(*n*)
Once a month or less	86	(80)	79	(223)
Several times a month or more	14	(13)	21	(61)
*χ* ^2^ (1) = 2.498 *p* > .05				

## Discussion

The physical job demands of surgeons were quantified for an average workday and compared with other hospital physicians. In comparison with other hospital physicians, surgeons perform fine repetitive movements 26 times longer and stand 130 % longer. In addition, more surgeons (41 %) find their work to be physically strenuous, are seriously bothered by making prolonged repetitive movements (35 %) and by working in uncomfortable and exhausting postures (73 %). A post hoc analysis revealed that the different gender distributions among surgeons and other hospital physicians did not influence these findings. The results bolster previous findings that surgeons contend with physical demands that are perceived as uncomfortable and exhausting (Kant et al. 1992). The presence of high physical demands has been considered to be a risk factor for developing physical health complaints (Stomberg et al. 2010). Whereas both surgeons and other hospital physicians experienced physical complaints mainly in the neck, arm or lower back region (prevalence rates ranging from 24 to 39 %), the majority of surgeons (50 % or more) who reported a physical complaint felt that their work was partly responsible for developing these complaints. In addition, a third of the surgeons (30 % or more) having a physical complaint in the arm and knee regions felt impaired in their work functioning. The majority of surgeons (86 %) reported that their physical state rarely affected their ability to cope with the physical job demands of their jobs; nevertheless, one out of every seven surgeons (14 %) regularly had difficulties coping with these demands due to impairments in their physical well-being. These findings constitute a warning that a number of surgeons are at risk for long-term sickness absence because of either reduced work ability or the presence of a physical health complaint (Roelen et al. 2007; Sell et al. 2009). Furthermore, reduced work ability is associated with reduced job performance and therefore poses a threat to the quality of care and, consequently, patients’ safety (Alavinia et al. 2009).

In this study, a representative sample from one population of surgeons and hospital physicians was used to gather information. With 51 % of the subjects completing the questionnaire, data about physical demands, physical health complaints and work ability are considered to be representative of the population. In addition, by following a measurement strategy for systematic observations that takes into account the variation in the frequency and duration of physical demands between and within workdays, the quantified physical demands are a reliable representation of the exposure to physical demands during an average workday. Altogether, it is justified to conclude that the physical demands of performing surgery are a threat to surgeons’ physical health, work ability and job performance. However, we cannot rule out over- or under response between the two groups and the generalization of these results might be restricted to other medical centers, while it is conceivable that surgeons in district hospitals might perform less difficult or complex operations.

To keep surgeons healthy on the job and to ensure a high quality of care, it appears necessary to take preventive measures that aim to reduce their physical strain. While job demands often cannot be easily reduced, a possible preventive measure would be to provide surgeons with sufficient recovery opportunities during the day. Empirical evidence shows that recovery from work is positively related to an employee’s health and well-being, as well as to job performance (Van Hooff et al. 2007; Binnewies et al. 2009). Currently, surgeons often lack recovery opportunities during surgery that could be achieved, for example, by a change in body posture. The lack of recovery opportunities becomes increasingly troublesome during an extensive surgical procedure. As a result, surgeons experience increased stress and fatigue throughout an operation, which may have an impact on the surgeon’s accuracy and the operation’s outcome (Slack et al. 2008). Providing on-the-job recovery opportunities during an operation, such as taking micro pauses or changing surgeons (Slack et al. 2008), could be an important prerequisite for not feeling strained or becoming fatigued and, instead, for performing well.

In reality, adopting awkward positions during difficult and prolonged surgical procedures is sometimes inevitable, and taking micro pauses or changing surgeons during a surgical procedure is impossible (Slack et al. 2008). In that case, circulating between tasks during a workday might provide additional recovery opportunities. Instead of performing several surgical procedures during one part of the workday, it is recommended that surgeons recover from surgery-induced physical strain by changing to less physically demanding tasks, such as ward rounds or report-writing, between surgical procedures. Finding ways to recover from physically strenuous work is important because chronic exposure to physically demanding work and incomplete recovery is an important pathway to chronic health impairment (Geurts and Sonnentag 2006).

In addition to exposure to high physical demands, the presence of high psychological job demands in combination with high physical demands has shown an even stronger relationship with the presence of physical complaints (Courvoisier et al. 2011). A high work-load with long working hours and a low decision latitude are examples of psychological job demands that surgeons and other hospital physicians experience (Arnetz 2001). Therefore, in addition to providing recovery opportunities for coping with the physical job demands, it is suggested that interventions are sought that aim to optimize the psychological work environment of surgeons, thereby reducing exposure to psychological job demands.

## Appendix 1

See Table 6.

**Table 6:** Hierarchical task analysis—physical variables of interest

Variable	Categories
Activities	Sitting Standing Walking Kneeling/squatting Working on a computer Walking the stairs Fine motoric movements Gross motoric movements Carrying Lifting Pushing/pulling
Body postures	Lumbar flexion (>60°)
	Lumbar rotation (>20°)
	Cervical flexion (>25°)
	Cervical rotation (>25°)
	Asymmetric posture
	One or two arms above shoulder height
	Reaching

## Appendix 2

See Table 7.

**Table 7:** Physical complaints reported in each body region for surgeons and other hospital physicians

Physical complaints	Surgeons (*n* = 91)		Hospital physicians (*n* = 281)	
	%	(*n*)	%	(*n*)
Neck	35	(32)	29	(82)
Work-related	84		67	
Work-impairing	13		15	
Upper back	19	(17)	14	(39)
Work-related	71		74	
Work-impairing	18		13	
Lower back	24	(22)	25	(69)
Work-related	59		38	
Work-impairing	18		16	
Shoulder	23	(21)	15	(43)
Work-related	71		61	
Work-impairing	48		23	
Elbow	4	(4)	4	(12)
Work-related	75		58	
Work-impairing	25		42	
Forearm	3	(3)	5	(14)
Work-related	67		57	
Work-impairing	33		43	
Wrist/hand	15	(14)	13	(36)
Work-related	57		53	
Work-impairing	57		31	
Hip	4	(4)	6	(17)
Work-related	25		0	
Work-impairing	25		6	
Knee	2	(2)	7	(20)
Work-related	50		15	
Work-impairing	50		10	
Leg	2	(2)	3	(7)
Work-related	100		0	
Work-impairing	0		43	
Ankle/foot	2	(2)	8	(23)
Work-related	50		17	
Work-impairing	0		17	
